# Mesenchymal stem cells in lung diseases and their potential use in COVID-19 ARDS: A systematized review

**DOI:** 10.1016/j.clinsp.2023.100237

**Published:** 2023-07-14

**Authors:** Bruna Benigna Sales Armstrong, Juan Carlos Montano Pedroso, José da Conceição Carvalho, Lydia Masako Ferreira

**Affiliations:** a Universidade Federal do Piauí (UFPI), Terezina, PI, Brazil; b Escola Paulista de Medicina (EPM), Universidade Federal de São Paulo (UNIFESP), São Paulo, SP, Brazil

**Keywords:** Mesenchymal stem cells, COVID-19, Lung disease

## Abstract

COVID-19 can converge with the pro-inflammatory immunoregulatory mechanisms of chronic lung diseases. Given the disorders inherent to lung transplantation and the inexistence of other definitive therapeutic alternatives, Adipose tissue-derived Stem Cells (ASCs) presented themselves as a therapeutic hope. The purpose of this review is to assess the basis for the potential use of ASCs in lung diseases unresponsive to conventional therapy, relating to their possible use in COVID-19 ARDS. 35 studies comprised this review, 14 being narrative reviews, 19 preclinical trials and two proofs of concept. COVID-19 can converge with the pro-inflammatory immunoregulatory mechanisms of chronic lung diseases. In view of the disorders inherent to lung transplantation and the inexistence of definitive therapeutic alternatives, Adipose tissue-derived Stem Cells (ASCs) presented themselves as a therapeutic hope. Its detailed reading indicated the absence of serious adverse effects and toxicity to the administration of ASCs and suggested possible effectiveness in reducing lung damage, in addition to promoting the recovery of leukocytes and lymphocytes with its immunomodulatory and anti-apoptotic effects. The revised clinical data suggests optimism in the applicability of ASCs in other immunoinflammatory diseases and in severe COVID-19 ARDS. However, further studies are needed to develop a consensus on the methods of collection of ASCs, the ideal dosage schedule, the most effective time and route of administration, as well as on the definition of indications for the administration of ASCs in cases of COVID-19 for conducting clinical trials in near future.

## Highlights

• None of the analized studies related serious adverse effects or toxicity to IV ASCs administration.
• This review suggests optimism in IV ASCs for lung damage in severe COVID-19 ARDS.
• Further studies on IV ASCs in COVID-19 are needed for standard dosage.

## Introduction

The end of 2019 was marked by the growing number of cases of severe respiratory illnesses of unknown origin in Wuhan, China; in January 2020, its etiologic agent, the contagious Severe Acute Respiratory Syndrome Coronavirus-2 (SARS-CoV-2) was identified [1]. Two months later, in March 2020, the World Health Organization (WHO) elevated a category of the 2019 Coronavirus Disease (COVID-19) from epidemic to the first pandemic caused by coronavirus, which on March 2, 2021 already illustrated a scenario with 2.6 million new confirmed and an increase of 63,000 deaths in the last week [2].

SARS-CoV-2 is one of three coronaviruses that evolve with Acute Respiratory Distress Syndrome (ARDS) [3]. Despite the genomic similarity of 79% to the Severe Acute Respiratory Syndrome Coronavirus (SARS-CoV) and 50% to the Middle East Respiratory Syndrome coronavirus (MERS-CoV), SARS-CoV-2 does not stand out for its relatively low 6.76% mortality, compared to 9.6% for SARS-CoV and 35.5% for MERS-CoV, but rather due to its high infectivity, which underscores the superiority of absolute numbers over percentage data [4].

Despite different etiologies, the pathophysiology of COVID-19 may converge to the same pro-inflammatory immunoregulators of chronic lung diseases:[3] abnormal repair processes with concomitant destruction of airway epithelium[5] and vascular endothelium [6]. However, regardless of the steady growth in the prevalence of asthma and Chronic Obstructive Pulmonary Disease (COPD) in recent years as well as COPD ranking third among the causes of chronic disease mortality worldwide, lung transplantation is still the only curative therapy for chronic lung disorders [1].

Due to the lack of other definitive therapeutic alternatives for chronic lung diseases and the disorders inherent to lung transplantation ‒ high donor incompatibility, lifelong need for immunosuppressive therapy, and high mortality rate after the procedure (50% in 5 years)[1] ‒ Preclinical and clinical studies of Mesenchymal Stem Cells (MSCs), with their paracrine immunomodulatory mechanisms that reduce pulmonary inflammation and promote tissue repair, have raised expectations about this possibility of treatment for chronic lung disease [1,7].

Even though, since their first description in 1968 [8], the number of clinical trials using MSCs in the management of lung diseases was somewhat unimpressive until this year, when the SARS-CoV-2 pandemic led to the pursuit of possible effective treatments, as of March 9, 2021, of the 110 studies registered in the National Institutes of Health (NIH) Clinical Trial Database on the use of cell therapy in lung diseases, 72 are specifically for COVID-19, with new studies being registered daily [9,10].

Adipose Tissue (TA) MSCs have received increasing attention over the years, both for their practical collection using local anesthesia [11], and for the greater quantity and easy isolation of target stem cells compared to those originating from Bone Marrow (BM) [11]. As one of the cellular components of the stromal Vascular Fraction (FVE), the portion of subcutaneous fat, it can be easily isolated by enzymatic degradation of adipocytes and cell expansion [11].

Although the analysis of experimental studies by Wecht and Rojas 12] has suggested both efficacy – reducing inflammation, preventing the progression of fibrosis, and accelerating tissue repair – and safety in the use of MSCs in chronic lung diseases, the effects of ASCs are underreported. Therefore, the objective of this study is to evaluate, through a systematic review of the literature, the therapeutic rationale of ASCs in chronic or acute pulmonary diseases that are unresponsive to conventional therapy, relating to their possible use in ARDS by COVID-19.

## Method

### General information

The present study is a systematized review of the literature. Systematized review is a classification described in the literature that attempts to include elements from the systematic review process to the narrative review while maintaining greater freedom in the quality assessment and comprehensive searching, all of which are shown in their limitations of methodology. To this end, the present article used an adaptation of the PRISMA guidelines suitable for systematized reviews.

The following databases were searched:

• CENTRAL (Cochrane Library) - https://www.cochranelibrary.com/
• CLINICAL TRIALS - https://clinicaltrials.gov
• LILACS (BIREME) - http://brasil.bvs.br/
• MEDLINE (PubMed) - https://www.ncbi.nlm.nih.gov/pubmed/
• SCOPUS - https://www.scopus.com
• WEB OF SCIENCE - https://www.webofscience.com

gray literature was also searched: http://www.opengrey.eu/ and https://www.worldcat.org/.

The descriptors (DeCS/MeSH) selected, in Portuguese and English, were: mesenchymal stem cells (células tronco mesenquimais), pneumonia (broncopneumonia) and pulmonary fibrosis (fibrose pulmonar).

### Search strategies

1 - ((pulmonary fibrosis[M*e*SH T*erms*]) OR (fibrose pulmonar [DeCS Terms]) OR (pneumonia[M*e*SH T*erms*]) OR (broncopneumonia[D*e*CS T*erms*])) AND ((mesenchymal stem cells[M*e*SH T*erms*]) OR (células tronco mesenquimais [DeCS Terms))

2 - Articles referenced by the works filtered from the search strategy that covered the eligibility criteria were also added.

### Selection process according to the inclusion and exclusion criteria

Publications were selected using the search strategy previously described, without date or language limitation. Duplicates and titles not related to the topic were excluded before the screening.

The inclusion criteria choice was based on the PICO strategy. The study population included lung diseases, the intervention analyzed was the infusion of mesenchymal stem cells derived from adipose tissue, which was compared to conventional treatment or placebo saline infusion and analyzed for efficacy and safety.

In the first selection process abstracts were reviewed for the following inclusion criteria: (a) Administration of Intravenous (IV) ASCs, which (b) Were not used as a concurrent vehicle for other therapeutic agents, as (c) Treatment for acute or chronic lung diseases.

The second selection process excluded: a) Editorials, comments, and letters to the editor, in addition to articles that b) Discussed exclusively non-adipose stem cells and derivatives, or that c) Did not involve the intravenous administration of ASCs in d) Pulmonary immunoinflammatory diseases.

### Endpoints

The evaluated outcomes can be divided according to two main approaches: efficacy and safety. The primary endpoint of the efficacy assessment was clinical parameters, while the primary endpoints of the safety assessment were descriptions of serious adverse events and death correlated to the intravenous administration of ASCs. Secondary outcomes included: a) For efficacy ‒ analysis of the homing capacity of ASCs, serial imaging tests, histopathology, cytology, biochemistry, TUNEL method, PCRs, and immunohistochemistry, in addition to taking into account the study design, its participants, the origin of ASCs and dosage administered for comparative purposes; as well as b) Safety ‒ mild adverse effects (transient fever, diarrhea, bronchitis and common colds) secondary to the IV infusion of ASCs.

## Results

**Fig. 1:**
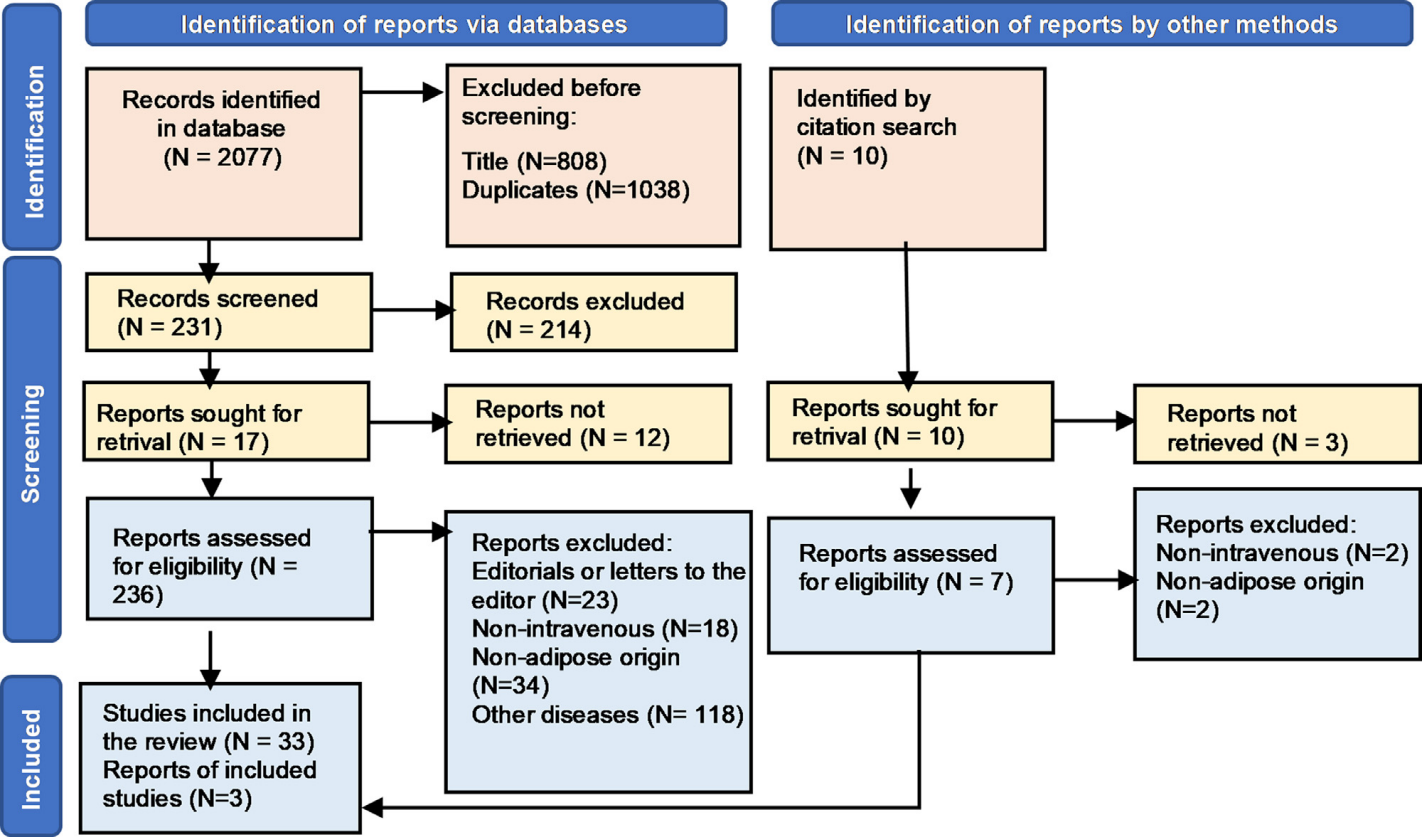
Flowchart of the selection process for researched articles. Legend: After inserting the search strategy in the databases, 2077 results were obtained, among which 1846 studies were initially excluded and only 231 articles were pre-selected, based on the reading of titles and abstracts. After evaluating the full text according to the eligibility criteria already described, 36 studies composed this review, being: 14 narrative reviews, 19 preclinical trials and three proofs of concept (N, Number).

After inserting the search strategy in databases, 2077 results were obtained, among which 1046 studies were initially excluded, then, based on the reading of titles and abstracts before the screening, only 231 articles were pre-selected (Fig. 1). After evaluating the full text according to the eligibility criteria already described, 36 studies composed this review, being: 14 narrative reviews, 19 preclinical trials and three clinical trials. The clinical characteristics of these studies are summarized in Tables 1, 2 and 3.

**Table 1:** Narrative reviews on the administration of ASCs in chronic or acute lung diseases.

Authors	Pathology	Effectiveness analysis				Safety analysis	
		Study method	Participants	Intervention	Results observed	Serious adverse events	Light adverse events
Barczyk et al. 2015	IPF	• Narrative review	Mices	• Tzouvelekis: autologous	Cell therapy for IPF appears to be overestimated based on currently available information.	None on IV infusion of ASCs. Tzouvelekis: Worsening of dyspnea: *n* = 2 (14%).	Transient fever tzouvelekis:
				• Lee: xerogenic			Fever: *n* = 7 (50%).
		• NR number of tests used in its preparation (16 in the table in the article exclusively about 1 of the 5 models of pulmonary fibrosis induction and 2 clinical tests in humans/ presented 159 references in all)		• Administration IV and EB		Oxygen desaturation: *n* = 2 (14%).	Cough worsening; *n* = 2 (14%).
				• Tzouvelekis: 0.5 × 10^6^/Kg, 3 doses with monthly intervals			
		• NR number of articles with ASCs (2 present in the table on pulmonary fibrosis induced by BLM and also presents 4 references that directly cite ASCS)		• Lee: 4 doses of 1 × 10^6^ applied concurrently with BLM			
		• Analysis of histopathology, biochemistry and immunohistochemistry					
		• Stem cell markers: (+) CD44, CD29, CD105 and CD90 and (-) CD45 and CD34					
Srour e Thébaud 2015	BLM-induced pulmonary fibrosis (PF)	• Narrative review	Mices	• Culture-expanded human adipose-derived xerogenic MSCs	MSC therapy was effective in animal models of BLM-induced lung injury. Most studies examined the early inflammatory phase providing a better representation of acute disease exacerbations.	None	Transient Fever
		• 17 studies used in its preparation					
		• 2 articles with ASCs (but only one IV)		• Dose: 0.3 × 10^6^ cells/kg IV (4 doses in weeks 8, 10, 12, and 14)			
		• Analysis of histopathology, collagen deposition, mortality, Aschcrott score and inflammatory markers: TGF-b, TNF-α, IFN-γ, IL6, IL1, MMP2, MMP9, MMP13					
		• The review does not describe the stem cell markers of the reviewed studies (CD)					
Stabler et al. 2015	Chronic lung diseases (ARDS, asthma and exposure to cigarette smoke)	• Narrative review	Guinea pigs and felines; ARDS patients	• Culture-expanded adipose-derived allogeneic MSCs	MSC-based therapies were effective and phase 1 clinical trials proved the safety of MSC therapy in ARDS, asthma, and exposure to cigarette smoke.	None	None
		• 20 studies used in its preparation					
		• 3 articles with ASCs (3 pre-clinical and 1 clinical)		• Zheng: 1 × 10^6^ cell/kg IV (DU)			
		• Analysis of the ability to differentiate clinical effects, anti-inflammatory effects and safety		• Preclinical: NR dosage			
		• The review does not describe the stem cell markers of the reviewed studies (CD)					
Geiger et al. 2017a	FPI; Acute respiratory distress syndrome, Chronic obstructive pulmonary disease	• Narrative review	NR	• Allogeneic and autologous MSCs derived from adipose tissue, expanded by culture	MSC-based therapies for pulmonary diseases present themselves as potential viable treatment options for clinical application. In particular, the potential of genetically modified MSCs, which allows for a considerable increase in therapeutic activity.	NR (Not reported)	NR
		• 24 tests (clinical and pre-clinical)					
		• 3 tests with ASCs (2 clinical and 1 pre-clinical)					
		• Analysis of lung function and safety		• Administered intravenously and EB			
		• The review does not describe the stem cell markers of the reviewed studies (CD)		• Phase I: 1 × 10^6^ cell/kg			
				• Phase Ib: 5 × 10^5^ MSC·kg-1			
				• Pre-clinical: 40 × 10^6^ MSCs·kg-1			
				• NR number of doses			
Antoniou et al. 2018	FPI; ARDS, COPD, severe emphysema, advanced pulmonary sarcoidosis	• Narrative review	Patients with ARDS	• Adipose tissue-derived, culture-expanded allogeneic MSCs	Recent clinical studies of the administration of autologous or allogeneic MSCs in patients with various lung diseases provide adequate evidence for the safety of using MSCs in these patient groups	NR (Not reported)	None
		• 8 clinical tests					
		• 2 tests with ASCs					
		• Analysis of pulmonary inflammation markers in IV administration		• Administered via IV and EB			
				• Single dose of 1 × 10^6^ cell./kg			
		• The review does not describe the stem cell markers of the reviewed studies (CD)					
Harrell et al. 2019	Immunoinflammatory lung diseases (ARDS, pneumonia, asthma, COPD, IPF)	• Narrative review	NR	• MSCs (does not say whether autologous or allogeneic) derived from adipose tissue, placenta, umbilical cord and culture-expanded bone marrow	The reviewed clinical trials suggest that the administration of MSCs was well tolerated and that MSC-based therapy is a safe therapeutic approach, as only a limited number of side effects have been reported.	None	Bronchitis and common cold were the most frequent
		• NR number of studies used in its preparation, but presented 119 references					
		• 4 articles with ASCs (1 clinician and 1 preclinical)					
		• Analysis of markers of lung inflammation, improvement in quality of life, lung function and safety		• Zheng: 1 × 10^6^ cell./kg IV (DU)			
				• Other ASCs: NR			
		• The review does not describe the stem cell markers of the reviewed studies (CD)					
Zanoni et al. 2019	Radiation-induced lung injury (LP)	• Narrative review	Humans and Mices	• NR origin (auto, alo, xero) of adipose stem cells	The lack of standardized methods for collecting MSCs and little or no information available on optimal dosage, timing and route of administration make it difficult to imagine the use of MSC-based therapy in clinical practice in the near future.	NR (Not reported)	NR
		• NR total number of studies used in its preparation (has 203 references)					
		• NR number of articles with ASCs (12 references cite ASCS directly)		• Administration IV and EB			
		• Analysis of histopathology, biochemistry and immunohistochemistry		• NR dose			
		• Stem cell markers: (+): CD105 (endoglin, SH2), CD73 (ecto-50-nucleotidase) and CD90 (Thy1) | (-): CD45, CD19 or CD79, CD14 or CD11b, and HLA-DR....					
Behnke et al. 2020	Bronchopulmonary dysplasia, Asthma, acute lung injuries (systemic and infectious), Chronic obstructive pulmonary disease (COPD)	• Narrative review	Humans and Mices	ORIGIN ADIPOSE STEM CELL PRE CLINICOS: • Cigarette smoke: (human × rat, human) • Elastase: mouse • Asthma: human • ALI: Humans • COPD: human • BLM: mices	The preclinical results raise high hopes that MSC-based therapies will successfully lead to cures rather than just relief of disease symptoms. Available data from clinical trials have proven the safety of such an age- and disease-entity approach.	In preclinical reports, there was death from DIC and cardiac and respiratory dysfunction due to the infusion of high doses of MSCs.	None
		• 75 studies used in its preparation					
		• 12 articles with ASCs [only 8 IVs: 1 from asthma, 1 from ALI, 1 from COPD, 3 from BLM, 2 from cigarette smoke (1 of them smoke or elastase) and 1 elastase (compare IV with IT)]					
		• Analysis of histopathology, biochemistry and immunohistochemistry		PRECLINICAL DOSE: • Cigarette smoke: 1 study used 3 × 10^5^ in 4 doses (weeks 8, 10, 12 and 14) and the other used 1 × 10^5^ DU • Elastase: 1 × 10^5^ DU • Asthma: 1 × 10^5^ DU • ALI: 1 × 10^6^ DU • COPD: 1 × 10^6^ DU • BLM: 2 studies used 5 × 10^5^ DU / and 1 study used 4 × 107 in 3 doses (days 3, 6 and 9)			
		• The review does not describe the stem cell markers of the reviewed studies (CD)					
				CLINIC • NR dose, neither if it was autologous or allogeneic.			
Cruz e Rocco 2020b	Chronic lung diseases (Asthma, COPD, Idiopathic pulmonary fibrosis-IPF, PAH, silicosis)	• Narrative review	Humans and Mices	• NR origin (auto, alo, xero) of adipose stem cells	MSC-based therapy is a promising alternative for the treatment of chronic lung diseases. Preclinical studies with MSCs generated great enthusiasm for their therapeutic potential in these conditions. Early clinical trials demonstrated that MSC administration is safe, with few adverse effects	None	None
		• NR total number of studies used in its preparation (it has 99 references)					
		• NR number of articles with ASCs (10 references cite ASCS directly)		• Administration IV			
		• Analysis of histopathology, biochemistry and immunohistochemistry		• NR dose			
		• Stem cell markers: (+) CD105, CD73, and CD90 and (-) CD45, CD34, CD14 or CD11b, CD79 alpha, or CD19, and HLA-DR....					
Ntolios et al. 2020	IPF	• Narrative review	Patients with mild to moderate IPF	• Allogeneic MSCs derived from adipose tissue, placenta and culture-expanded bone marrow	Clinical trials currently completed suggest that cell therapies are safe and can be effective	None	Phase Ib EB: minor adverse effects, mainly related to bronchoscopy.
		• 9 clinical tests					
		• 3 tests with ASCs (12, 15, 60 pcts)					
		• Clinical and radiological analysis					
		• Safety and laboratory analysis of inflammatory markers: C-reactive protein, LDH, d-dimer and ferritin		• Administered intravenously and endobronchial			
		• Stem cell markers (+): CD105, CD73, CD90, CD44, CD71, Stro1, CD106 (VCAM-1), CD166 (ALCAM), ICAM-1, CD29; and (-): CD45, CD34, CD11, CD80, CD86, CD40, CD31 (PECAM-1), CD18, CD56, HLA II					
				• 1 Phase I: 1 × 10^6^ cells/kg IV (DU)			
				• 2 Phase Ib: 5 × 10^5^ cell./kg EB (3 doses 1 month apart)			
Qin e Zhao 2020	ARDS and COVID-19	• Narrative review	NR	• MSCs (does not say whether autologous or allogeneic) derived from adipose tissue, placenta and culture-expanded bone marrow	The safety of MSC therapy has been demonstrated in early-stage clinical trials with a small number of patients. Systemic administration of MSC proved to be effective.	None	None
		• 18 tests (clinical and pre-clinical)					
		• 2 tests with ASCs (1 clinician and 1 preclinical)					
		• Analysis of markers of lung inflammation, onset of antimicrobial response, protective effects, decrease in damage to distal organs		• Clinical: 1 × 10^6^ cells/kg IV (DU)			
				• Pre-clinical: NR			
		• The review does not describe the stem cell markers of the reviewed studies (CD)					
Rogers et al. 2020a	ARDS and COVID-19	• Narrative review	Mices and humans	• NR reported dose	Cell-based therapies have demonstrated safety in human clinical trials, warranting further investigation	None	Transient Fever
		• NR total number of studies used in its preparation		REFERRED DOSE:			
		• NR number of articles with ASCs		• Perlee: 4 × 10^6^ ASCs/kg (NR number of doses)			
		• Analysis of histopathology, biochemistry and immunohistochemistry		• Zheng: 1 × 10^6^ DU			
		• The review does not describe the stem cell markers of the reviewed studies (CD)					
Yen et al. 2020b	Immunoinflammatory lung diseases (ARDS, COPD, IPF…)	• Narrative review	NR	• Allogeneic and autologous MSCs derived from adipose tissue, expanded by culture	MSC for COVID-19 should be targeted to very severe cases where ARDS and an exuberant immune response are observed. Preclinical MSC data were quite consistent, and MSC clinical data in other immunoinflammatory diseases support the relative safety of MSC therapy, even though the efficacy may be more difficult to interpret.	NR (Not reported)	NR
		• 68 clinical trials					
		• 12 tests with ASCs					
		• Does not report the analysis parameters of the results					
		• The review does not describe the stem cell markers of the reviewed studies (CD)		• Does not report via Administration			
				• Does not report dose schedule			
Xiao et al. 2020	ARDS and COVID-19	• Narrative review	Patients with ARDS	• MSCs (does not say whether autologous or allogeneic) derived from adipose tissue, menstrual blood, umbilical cord and culture-expanded bone marrow	Safety and possible efficacy have been demonstrated in some patients with ARDS. Although some progress has been made, there is insufficient clinical evidence to prove the efficacy of MSCs in treating ARDS.	None	None
		• NR number of studies used in its preparation, but presented 48 references					
		• 1 articles with ASCs (clinical)					
		• Analysis of markers of lung inflammation, clinical improvement and safety					
				• Zheng: 1 × 10^6^ cell/kg IV (DU)			
		• The review does not describe the stem cell markers of the reviewed studies (CD)					

COVID-19, 2019 Coronavirus Disease; ARDS, Acute Respiratory Distress Syndrome; COPD, Chronic Obstructive Pulmonary Disease; IPF, Idiopathic Pulmonary Fibrosis; PAH, Pulmonary Arterial Hypertension; PF, Pulmonary Fibrosis; BLM, Bleomycin; LP, Lung Lesion; NR, Does Not Refer; ASCs, Adipose tissue-derived Stem Cells; TGF-b, Transforming Growth Factor beta; TNF-α, Tumor Necrosis Factors Alpha; IFN-γ, Interferon-gamma; IL, Interleukin; MMP, Metalloproteinases; IV, Intravenous; IT, Intratracheal; EB, Endobronchial; DU, Single Dose; kg, Kilogram; MSC, Mesenchymal Stem Cells; CD, Differentiation Cluster; cell., Cells.

**Table 2:** Preclinical trials on the administration of ASCs in chronic or acute lung diseases.

Authors	Pathology	Effectiveness analysis					Safety analysis	
		Study method	Participants	Intervention	Follow-up	Results observed	Serious adverse events	**Light adverse events**
Schweitzer et al. (2011)	Cigarette smoke-induced lung injury (LP)	• Pre-clinical	• Mices	• Allogeneic MSCs derived from adipose tissue of animal and xerogenic (human) origin, expanded by culture	• Follow up of lung tissue: 1, 7, and 21 days after administration	The results suggest a useful therapeutic effect of adipose stem cells in both lungs and systemic injury induced by cigarette smoke and imply a pulmonary vascular protective function of paracrine factors derived from adipose stem cells.	NR (Not reported)	NR
		• 20 mices						
		• NR randomization of group division						
		• Biochemical and immunohistochemical analysis						
				• Administration IV				
		• Inflammatory markers: caspase 3, via MAPK		• Single dose: 3 × 10^5^ cells ASCs (in both experiments)				
		• Stem cell markers: anti-CD31b						
Gao et al. (2013)	Acute Lung Injury (ALI)	• Pre-clinical	• Mices	• Xerogenic MSCs derived from human adipose tissue, expanded by culture	• Follow up: The culture medium was collected at 24 h, 48 h and 72 h; rat plasma was collected in 7 days.	ASCs were able to attenuate the severity of ALI and pulmonary edema.	NR (Not reported)	NR
		• 25 Mices						
		• RCT - control group (10)						
		• Clinical, biochemical, immunohistochemical and wet-dry lung ratio analysis		• Administration IV				
				• Single dose MSC: ∼5 × 10^5^ ASC				
		• Inflammatory marker: NO						
		• Stem cell markers: PE-CD34, FITC—CD90 and PE-106 antibodies						
Cho et al. (2014)	Asthma	• Pre-clinical	• Mices	• Allogeneic MSCs derived from adipose tissue of animal origin, expanded by culture	• Follow up: airway hyperresponsiveness was assessed on day 23. The frequency of sneezing and nasal rubbing that occurred within 10 min of the last ovalbumin administration (day 23). The mices were euthanized on day 24. At least 48 h after the last OVA administration, serum was collected from the mices.	IV ASCs significantly reduced allergic symptoms and inhibited eosinophilic inflammation.	NR (Not reported)	NR
		• 20 mices						
		• NR randomization of group division						
		• Clinical, biochemical, immunohistochemical and histopathological analysis		• Administration IV				
				• 4 Doses: 1 × 10^7^/mL ASC cells suspended in PBS (days 12, 13, 19 and 20)				
		• Inflammatory markers: IL-4, IL-5, IL-10, IL-13, IFN-γ, TGF-β, Ig E, IgG1, and IgG2a, PGE2, IDO enzyme						
		• Stem cell markers: (+): Sca1, CD44, CD90; (-): CD45, CD 117 and CD11b						
Feizpour et al. (2014)	COPD	• Pre-clinical	• Guinea pigs	• Allogeneic cryopreserved MSCs derived from adipose tissue of animal origin, expanded by culture	• Follow-up: 14 days	No significant changes were observed in the group that received ASCs IV.	NR (Not reported)	NR
		• 36 guinea pigs						
		• RCT- control group (6 via IT and 5 via IV)						
		• Tracheal, biochemical and cytological responsiveness analysis		• Administration IV and IT				
		• Inflammatory markers: IL-8		• Single dose: 0.3 mL PBS containing 10^6^ ASCs (both lanes)				
		• Stem cell markers: feline anti-CD4 PE, anti-feline CD5 biotin and streptavidin APC						
Lee et al. (2014)	BLM-induced pulmonary fibrosis (PF)	• Pre-clinical	• Mices	• Culture-expanded xerogenic adipose tissue-derived MSCs	• Follow-up: mices were euthanized on day 16. Lungs were collected 2 weeks after the last dose of ASCs that occurred on day 14.	BLM-ASC treatment resulted in a significant decrease in the number of apoptotic and inflammatory cells, as well as a reduction in fibrosis score compared to group only with BLM.	NR (Not reported)	NR
		• 40 mices						
		• Did not describe the method of dividing the groups, whether it was randomized or not (control: *n* = 10)		• Administration IV				
				• 4 Doses (1 every 2 weeks for 2 months) single: 3 × 10^5^ ASCs				
		• Cytological, histological, immunohistochemical and TUNEL method analysis						
		• Inflammatory markers: TGF-b						
		• Stem cell markers: (+): CD73 and CD105; (-): CD14, CD34 and CD45						
Kim et al. (2014)	Elastase-induced pulmonary emphysema	• Pre-clinical	• Mices	• Xerogenic MSCs derived from human adipose tissue, expanded by culture	• Follow-up: Mices were euthanized after 1, 4, 24, 72 and 168 h.	The results show that injected MSCs were observed 1 and 4 h after injection and more MSCs remain in the emphysema lungs.	NR (Not reported)	NR
		• NR the number of Mices						
		• NR randomization of group division						
				• Administration IV				
		• Image and molecular analysis (PCR)		• Single dose: 5 × 10^5^ ASCs in 100 μL saline				
		• Image analysis after 1, 4, 24, 72 and 168 h.		• Stem cell markers: NR				
Trzil et al. (2014)	Asthma	• Pre-clinical	• Cats	• Allogeneic cryopreserved MSCs derived from adipose tissue of animal origin, expanded by culture	• Follow-up: Allergen challenges were performed weekly for 4 months after the first infusions. Subsequent challenges were performed bimonthly between months 4 and 8 and monthly from 8 months until the end of the study.	When given after the development of feline chronic allergic asthma, MSCs have failed to reduce airway inflammation. However, repeated administration of MSCs at baseline reduced airway remodeling at month 8 CT, although it was not maintained at month 12.	∼1 month after study completion, one cat developed an aggressive sarcoma. post-death exam confirmed spindle cell sarcoma without evidence of other malignant or metastatic disease.	None
		• 9 cats						
		• RCT- control group (4)						
		• Clinical analysis, biochemistry, immunohistochemistry, cytology and imaging		• Administration IV				
				• 6 doses (2 × /month): 3.64 × 106 to 2.50 × 10^7^ MSCs (average of 1.44 × 107 MSCs alive / infusion)				
		• Inflammatory markers: IL10, IgE, lymphocytes and eosinophils in BALF						
		• Company-proven stem cells						
Dong et al. (2015)	Radiation-induced lung injury (LP)	• Pre-clinical	• Mices	• Xerogenic MSCs derived from human adipose tissue, expanded by culture	• Follow-up: Mices were euthanized on day 3, after 1 week, 2 weeks, 4 weeks, 12 weeks and 24 weeks to perform the necessary analyses.	The results confirmed that mesenchymal stem cells have the potential to limit pulmonary fibrosis after exposure to ionizing irradiation.	NR (Not reported)	NR
		• First part: 108 Mices						
		• Second part: 48 mices						
		• First part control: 12 (did not specify group division technique)		• Administration IV				
				• Single dose: 5 × 10^6^ ASCs (2 h after irradiation)				
		• Control second part: 27 mices (did not specify group division technique)						
		• Biochemical, immunohistochemical and histopathological analysis						
		• Inflammatory markers: TGF-β1, TNF-α, PGE2, HGF, IL-10, COX1 enzyme, COX2 enzyme and IGF						
		• Stem cell markers: CD11b, CD19, CD34, CD45, CD73, CD90, CD105 and HLA-DR....						
Fikry et al. (2015)	MTX-induced pulmonary fibrosis (PF)	• Pre-clinical (comparative)	• Mices	• Allogeneic MSCs derived from adipose tissue and culture-expanded rat bone marrow.	• Follow-up: mices were euthanized after 6 weeks	Both BM-MSCs and ASCs exerted antifibrotic effects on MTX as a model of pulmonary fibrosis, which can be attributed to their antioxidant and anti-apoptotic properties, therefore, they can be presented as promising candidates for the treatment of pulmonary fibrosis.	NR (Not reported)	NR
		• 40 mices						
		• RCT - control group (8)						
		• Biochemical, immunohistochemical and histopathological analysis		• Administered intravenously				
				• Low dosage: 2 × 10^6^ cel.				
		• Inflammatory and oxidative stress markers: IL4, TGF-b1, (MDA, GSH, SOD).		• High dosage: 4 × 10^6^ cel				
		• Stem cell markers (+): CD90 and CD105 and (-): CD34						
Perlee et al. (2019a)	Pneumossepsis caused by Klebsiella pneumoniae	• Pre-clinical	• Mices	• MSCs derived from adipose tissue not reported origin, expanded by culture as well as cryopreserved	• Follow-up: Analyzes were performed after euthanasia. Mices infused with ASCs 1 h after infection were sacrificed 4 h or 16 h after pneumonia induction; mices infused with ASCs 6 h after infection were sacrificed 48 h after pneumonia induction.	These data indicate that ASC-associated tissue factor is responsible for systemic activation of coagulation after ASC infusion, but not for the formation of microthrombi in the lungs or for the antibacterial effects.	NR (Not reported)	NR
		• Mices (does not say total amount)						
		• Has a control group, but does not specify group division methodology (4‒8)						
				• Administration IV				
				• High single dose: 1 × 10^6^ ASCs (1 or 6 h after infection)				
		• Biochemical, immunohistochemical and histopathological analysis		• Low single dose: 0.4 × 10^6^ cells, 6 h after infection				
		• Inflammatory markers: Fibrin						
		• Stem cell markers: CD32, CD45 mAb, CD90 mAb, CD16						
Perlee et al. (2019b)	Pneumossepsis caused by Klebsiella pneumoniae	• Pre-clinical	• Mices	• Allogeneic MSCs derived from adipose tissue not reported origin, expanded by culture	• Follow-up: Mices were euthanized 16 or 48 h after pneumonia infusion	Both cultured and cryopreserved ASCs were able to reduce bacterial growth and dissemination during K. pneumoniae-induced pneumosepsis, with cryopreserved cells exerting a faster effect at the primary site of infection and with a dose-dependent effect.	NR (Not reported)	NR
		• 50 mices						
		• RCT ‒ control group (10)						
		• Biochemical, immunohistochemical and histopathological analysis		• Administration IV				
				• High single dose: 1 × 10^6^ ASCs, 1 or 6 h after infection				
		• Inflammatory markers: IL-1β, IL-6, TNF-α, MIP-2, MPO, E-selectin, VCAM-1 and MCP-1		• Low single dose: 0.4 × 10^6^ cel. 6 h after infection				
		• Stem cell markers: CD32, CD45 mAb, CD90 mAb, CD16						
Jiang et al. (2015)	Radiation-induced lung injury (LP)	• Pre-clinical	• Mices	• Allogeneic MSCs derived from rat adipose tissue, expanded by culture	• Follow-up: days 1, 3, 7, 14 and 28	ASCs reduced serum levels of pro-inflammatory cytokines, increased levels of anti-inflammatory and regulated the expression of pro- and anti-apoptotic mediators to protect lung cells.	NR (Not reported)	NR
		• 90 Mices						
		• RCT ‒ control group (30)						
		• Biochemical, immunohistochemical, localization (fluorescence microscopy) and histopathological analysis		• Administration IV				
				• Single dose: 5 × 10^6^ ASCs (2 h after irradiation)				
		• Inflammatory markers: IL-1, IL-6, IL-10, TNF-α, TGF-β1 and HGF						
		• Stem cell markers: CD11b-PE, CD29-PE, CD44-FITC and CD45-APC.						
Mao et al. (2015)	Acute Lung Injury (ALI)	• Pre-clinical	• Mices	• Allogeneic MSCs derived from adipose tissue of animal origin, expanded by culture	• Follow-up: 24 h after P. aeruginosa infection	ASCs exhibited protective effects against pulmonary P. aeruginosa infection.	NR (Not reported)	NR
		• NR number of Mices						
		• NR randomization and division of groups						
		• Clinical, biochemical, immunohistochemical and histopathological analysis		• Administration IV				
				• High single dose: ∼5 × 10^6^ ASCs				
		• Inflammatory markers: KGF, Ang-1, IGF-1, PGE2, COX2 and 15-PGDH		• Low single dose: ∼5 × 10^5^ ASCs				
		• Stem cell markers: (+): CD34, CD45; (-): CD90, CD105						
Tashiro et al. (2015)	BLM-induced pulmonary fibrosis (PF)	• Pre-clinical	• Mices	• Allogeneic MSCs derived from the adipose tissue of young mices, expanded by culture	• Follow-up: all mices were euthanized on day 21 for analysis.	The fibrosis score in the lungs of mices that received BLM was decreased in those treated with yASCs, however, the score in those treated with oASCs remained high.	NR (Not reported)	NR
		• Mices (did not say quantity)						
		• Has control (did not specify group division technique)		• Administration IV				
				• Single dose: 5 × 10^5^ ASCs				
		• Biochemical, immunohistochemical and histopathological analysis						
		• Inflammatory markers: TGF-β, integrin-αv, TNF-α, VEGF, Nrf2, MMP-2, ROS, and IGF						
		• Stem cell markers: CD90, CD205, CD29, Sca1, CD79α, CD45, CD14 and CD11						
Reddy et al. (2016)	BLM-induced pulmonary fibrosis (PF)	• Pre-clinical (comparative)	• Mices	• Xerogenic MSCs derived from human adipose tissue, subjected to enzymatic degradation	• Follow-up: all mices were euthanized on day 24 for analysis.	Survival was significantly prolonged and better in mices treated with ASC than pirfenidone. After the infusions, the disease characteristics disappeared significantly on day 21, it also demonstrated homing and graft potential towards the damaged lung tissue, being detected on day 24 after administration.	NR (Not reported)	NR
		• 50 mices						
		• RCT - control group (10)						
		• Radiological, biochemical, immunohistochemical and histopathological analysis		• IV administration, 3 doses (3 days between)				
				• Dose: 40 × 10^6^ cel./kg (equivalent in a human to 2 × 10^6^/kg)				
		• Inflammatory markers: IL2, IL1b, TNF-α, TGF β, bFGF, CTGF, CoL3a1, CoL1a1, MMP-TIMP						
		• Stem cell markers: CD34, CD45, CD73, CD90, CD105, CD166						
Pedrazza et al. (2017)	Acute Lung Injury (ALI)	• Pre-clinical	• Mices	• Allogeneic MSCs derived from adipose tissue of animal origin, expanded by culture	• Follow-up: After 7 days, animals that were still alive were anesthetized. Analyzes were performed 12 h after administration of ASCs.	The mices that received MSCs had a significantly higher survival rate compared to the LPS group, improvements in cytological, histological and biochemical analyses, indicating a possible action of MSCs via neutrophils.	NR (Not reported)	NR
		• NR total number of mices						
		• NR randomization or division of groups						
		• Cytological, histological, immunohistochemical and biochemical analysis		• Retro orbital IV administration				
				• Single dose: 5 × 10^5^/100 μL PBS				
		• Inflammatory markers: IL-6, TNF-α, IL-10, COX-2, GAPDH enzyme, NF-κB						
		• Stem cell markers: (+): CD73 and CD105; (-): CD14, CD34 and CD45						
Chen et al. (2018)	Silicosis-induced pulmonary fibrosis (PF)	• Pre-clinical	• Mices	• Allogeneic MSCs derived from rat adipose tissue, expanded by culture	• Follow-up: 28 days	Treatment with transplant ASCs led to a remissive effect on pulmonary fibrosis.	NR (Not reported)	NR
		• 20 mices						
		• RCT - control group (5)						
		• Biochemical, immunohistochemical and histopathological analysis		• Administration IV				
				• Single dose: 5 × 10^5^ ASCs (24 h after exposure to silica)				
		• Inflammatory markers: TNF-α, IL-1β, IL-6 and IL-10						
		• Stem cell markers: CD44, CD45, CD90, CD73 and CD11b						
Felix et al. (2020)	BLM-induced pulmonary fibrosis (PF)	• Pre-clinical	• Mices	• Allogeneic MSCs derived from adipose tissue of animal origin, expanded by culture and ASC-MC.	• Follow-up: 14 and 21 days	Mices that were injected with MSCs and MC showed improvement in general status, in addition to presenting an early anti-inflammatory action and improvement in fibrotic markers.	NR (Not reported)	NR
		• 40 mices						
		• RCT - control group (10)						
		• Clinical, biochemical, immunohistochemical and histopathological analysis		• Administration IV				
				• Single dose high MSC: 1 × 10^6^ ASCs in 0.2 mL of serum free medium (10 days after induction)				
		• Inflammatory and fibrotic markers: fibrinogen, Von Willebrand factor, PDGF, NOS, IL-17, TGF-β, VEGF, endothelin-1 and the immunogenic Col. V in lung tissue of mices with MBL lesion after treatment with MSCs		• DU MC: 200 μL, derived from 1 × 10^6^ cel. (10 days after induction)				
		• Stem cell markers: CD34, CD45, CD90						
Radwan et al. (2020)	Amiodarone-induced pulmonary fibrosis (PF)	• Pre-clinical	• Mices	• Allogeneic MSCs derived from culture-expanded rat adipose tissue	• Follow-up: At the end of 12 weeks in order to confirm induction of pulmonary fibrosis, three animals were randomly euthanized from the control and amiodarone-treated groups. After the end of the experimental period (2 months), all animals fasted for 12 h and blood samples were collected	Treatment with ASC resulted in improvement of biochemical and histopathological parameters.	NR (Not reported)	NR
		• 40 mices						
		• RCT - control group (10)						
		• Biochemical, immunohistochemical and histopathological analysis		• Administered intravenously				
				• Low dosage: 2 × 10^6^ cel.				
		• Inflammatory markers: CC16 protein, CK19 protein, αSMA.		• High dosage: 4 × 10^6^ cel.				
		• Stem cell markers (+): CD90 and CD105; and (-): CD34						

PF, Pulmonary Fibrosis; BLM, Bleomycin; MTX, Methotrexate; ALI, Acute Lung Injury; LP, Lung Injury; COPD, Chronic Obstructive Pulmonary Disease; RCT, Randomized Trial with a Control group; αSMA, α Smooth Muscle Actin; IL, Interleukin; TGF-β, Transforming Growth Factor Beta; TNF-α, Tumor Necrosis Factors Alpha; bFGF, Basic Fibroblast Growth Factor; CTGF, Connective Tissue Growth Factor; Col., Collagen; MMP, Metalloproteinases; VEGF, Endothelial Growth Factor; Nrf2, Factor 2 Related to Nuclear erythroid Factor 2; ROS, Reactive Oxygen Species; IGF, Insulin-Like Growth Factor; MDA, Malondialdehyde, GSH, Reduced Glutathione; SOD, Superoxide Dismutase; HGF, Hepatocyte Growth Factor; PG, Prostaglandin; MIP, Macrophage Inflammatory Protein, MPO, Myeloperoxidase; VCAM, Vascular Cell Adhesion Molecule; MCP, Monocyte Chemotactic Protein; PDGF, Platelet-Derived Growth Factor; NOS, Nitric Oxide Synthase; NO, Nitric Oxide; KGF, Keratinocyte Growth Factor; Ang-1, Angiotensin 1; PGDH, Hydroxyprostaglandin Dehydrogenase; IFN-γ, Interferon-Gamma; Ig, Immunoglobulin; IDO, Indoleamine 2,3 Dioxygenase; BALF, Bronchoalveolar Lavage; IV, Intravenous; IT, Intratracheal; GAPDH, Glyceraldehyde-3-Phosphate Dehydrogenase; MSC, Mesenchymal Stem Cells; ASC-MC, Conditioned Medium from in vitro Adipose Cell Culture; DU, Single Dose; cell., Cells; mL, Milliliter; µL, Microliter; CD, Differentiation Cluster; ASCs, Adipose issue-derived Stem Cells; kg, Kilogram; PE, Phycoerythrin; FITC, Fluorescein Isothiocyanate; APC, Antigen Presenting Cell; HLA, Human Leukocyte Antigen System; mAb, Monoclonal Antibodies; NR, Does Not Refer; yASCs, ASCs taken from young animals; oASCs, ASCs taken from elderly animals; BM-MSCs, Bone Marrow-derived Stem Cells; LPS, Lipopolysaccharide; NETs, Extracellular Neutrophil Traps; NR, Does Not Refer.

**Table 3:** Published clinical trials on the administration of ASCs in chronic or acute lung diseases.

Authors	Pathology	Effectiveness analysis					Safety analysis	
		Study method	Participants	Intervention	Follow-up	Results observed	Serious adverse events	Light adverse events
Zheng et al. (2014)	ARDS	• Single-center, randomized, double-blind, placebo-controlled trial.	• 12 Patients with ARDS aged at least 18 years and diagnosed within 48 h with a PaO2/FiO2 ratio of < 200.	• Follow-up: days 1, 3, 5, 7, 14 and 28 (or until hospital discharge or death, whichever comes first).	• Adipose tissue-derived allogeneic MSCs expanded by culture in patient serum	There were no infusion toxicity or serious adverse events related to MSC administration and there were no significant differences in the overall number of adverse events between the two groups.	None	One patient in each group had diarrhea one day after treatment resolved within 48 h. One patient in the MSC group developed a rash in the chest area after the infusion and resolved spontaneously over 24 h
		• 12 Patients			• Administered IV			
		• RCT - control			• DU: 1 × 10^6^ /kg.			
		• Primary endpoint: occurrence of adverse events. Secondary endpoints included the following: PaO2/FiO2 ratio, length of stay, days without ventilation, days without ICU on day 28, IL-6 and IL-8.	• Average age in the MSCS group: 66.7 years | in control: 69.8 years		• CD73, CD90, CD105, CD34, CD45 and HLA-DR....			
Leng et al. (2020)	SARS-COV-2	• Concept proof	• 7 patients with COVID (CRP +) and unresponsive to conventional therapies with persistent worsening of the condition	• Average follow-up: 14 days	• MSCs of undefined origin	No acute infusion-related or allergic reactions were observed within two hours of transplantation. Likewise, no delayed hypersensitivity or secondary infections were detected after treatment.	None	None
		• 7 Patients			• Administered intravenously			
		• RCT			• Single dose: 1 × 10^6^ /kg.			
		• 1st safety endpoint: secondary infection and life-threatening adverse events. 1st efficacy endpoint: level of variation in cytokines, serum C-reactive protein and oxygen saturation. 2nd efficacy endpoint: total lymphocyte and subpopulation count, chest CT, respiratory rate, patient symptoms, therapeutic measures and their results.			• Does not describe stem cell (CD) markers			
			• Ages ranging between: 45 and 75 years old					
Sánchez-Guijo et al. (2020)	COVID-19	• Concept proof	• 3 Patients with COVID-19 (CRP + Rx or CT) and on mechanical ventilation	• Average follow-up: 14 days	• Culture-expanded adipose-derived allogeneic MSCs	Treatment with ASC proved to be safe and resulted in a decrease in inflammatory parameters, as well as an increase in lymphocytes, especially in those patients with clinical improvement.	None	None
		• 13 Patients with COVID-19 (CRP + CX or chest CT) on mechanical ventilation						
					• Administered IV			
					• Average number of cells per dose: 0.98 (IQR 0.5) × 10^6^ /kg.			
		• No control group	• Average age: 60 years old					
		• Clinical and radiological analysis	• Average time between MSC dose and extubation: 7 days		• 1 pct: 3 doses; 2pcts: 2 two; 10 pcts: 2 doses			
		• Laboratory analysis of inflammatory markers: C-reactive protein, LDH, d-dimer and ferritin			• + CD90 and CD105; - CD34			

COVID-19, 2019 Coronavirus Disease; ARDS, Acute Respiratory Distress Syndrome; PCR, Reverse Transcription followed by Polymerase Chain Reaction; X-Ray, Radiography; CT, Computed Tomography; RCT, Randomized Trial; LDH, Lactate Dehydrogenase; FiO2, Inspired Oxygen Fraction; PaO2, Arterial Oxygen Pressure; ICU, Intensive Care Units; IL, Interleukin; MSC, Mesenchymal Stem Cells; IV, Intravenously; kg., Kilogram; pct(s), Patient(s); CD, Differentiation Cluster; HLA, Human Leukocyte Antigen system; DU, Single Dose; IQR, Interquartile Range; ASCs, Adipose tissue-derived Stem Cell; sCABP, Severe Community-Acquired Bacterial Pneumonia; IMV, Invasive Mechanical Ventilation.

The search in the clinical trials database resulted in 29 studies of adipose-derived stem cells in lung diseases, their official status being: one no longer available, five unknown, five withdrawn, one enrolling by invitation, four recruiting, four not yet recruiting, one suspended, two terminated, six completed. No study has published its results in academic journals in the literature to date. The population, intervention, comparator and outcome of these studies are summarized in Table 4.

**Table 4:** Unpublished clinical trials on the administration of ASCs in chronic or acute lung diseases.

Title (year)	Study method	Population	Intervention	Comparator	Outcomes	Status
Safety and Efficacy of Adipose Derived Stem Cells for Chronic Obstructive Pulmonary Disease (2014)	• Phase I/II Open-label, single group assignment, Non-Randomized, Multi-center Study	• 26 patients (Age 18 to 85, prior diagnosis of moderate to severe COPD; GOLD IIa, III, IV; Cognitive competitiveness; life expectancy > 6 months, written informed consent)	• 100‒240cc of lipoaspirate will be extracted from the patient. The SVF will be isolated with minimal manipulation. The cell pellet will be reconstituted in saline solution and administered intravenously to the patient as a single dose of autologous adipose derived stem cells. The dosage was not described	None	• Primary outcomes: FEV1 Decline [Time Frame: 12 months] and Number of Adverse Events [Time Frame: 12 months]	Completed
					• Secondary outcomes: Secondary Efficacy Objective [Time Frame: 12 Months]	
Safety, Tolerability and Preliminary Efficacy of Adipose Derive Stem Cells for Patients With COPD (2014)	• Phase I Open- Label, single group assignment, study to assess safety and tolerability	• 9 patients (males and females ≥18 years. Cognitive competitiveness. Diagnosis of at least moderate, COPD, Diffusing capacity impairment, assessed by single breath test, life expectancy > 12 months, written informed consent, non-smoker or past smoker, with 20 pack-years or more history)	• 100‒240cc of lipoaspirate will be extracted from the patient. The SVF will be isolated with minimal manipulation. The cell pellet will be reconstituted in saline solution and administered intravenously to the patient as a single dose of autologous adipose derived stem cells. The dosage was not described	None	• Primary outcomes: Safety of adipose derived stem cells (ADSC) in Patient with COPD [Time Frame: 12 months]	Terminated
					• Secondary outcomes: Efficacy of ADSC in improving Shortness of Breath (SOB) [Time Frame: 2, 6 and 12 months]; Efficacy of ADSC In Pulmonary Function Test (PFTs) [Time Frame: 2, 6, 12 months]; Efficacy of adipose derived stem cell in 6 MWT [Time Frame: 2, 6, 12 months]; Efficacy of adipose derived stem cells in patient's perceived exertion [Time Frame: 2, 6, 12 months]; Efficacy in Quality of life using George's Respiratory Questionnaire [Time Frame: 2, 6, 12 months]; Efficacy in Quality of life using the Chronic Respiratory questionnaires [Time Frame: 2, 6, 12 months].	
Adipose Derived Stem Cells Transplantation for Chronic Obstructive Pulmonary Disease (2016)	• Phase I/II open- label single-dose study in subjects with significant COPD.	• 20 patients (Age 40 to 80 + prior diagnosis of moderate to severe COPD GOLD IIa, III, IV)	• Autologous SVF and PRP will be transfused into 20 COPD patients.	None	• Primary outcomes: SGOT [Time Frame: 1 month], SGPT [Time Frame: 1 month]	Unknown
					• Secondary outcomes: Respiration rate [Time Frame: 1 month, 6 months, 12 months], 6 min walk test [Time Frame: 1 month, 6 months, 12 months],rates of panic attacks [Time Frame: 1 month, 6 months, 12 months], CRP concentration [Time Frame: 6 months, 12 months].	
Adipose Derived Cells for Chronic Obstructive Pulmonary Disease (2014)	• Open-label, Non-Randomized, Multi-Center Study to Assess the Safety and Effects	• 0 patients	• Adipose Derived Stem Cells. The dosage or origin was not described	None	• Primary outcomes: assess safety	Withdrawn
					• Secondary outcomes: efficiency in improving the disease pathology of patients with diagnosed with chronic obstructive pulmonary disease	
Safety and Efficacy of Adipose Derived Stem Cells for Chronic Obstructive Pulmonary Disease (2012)	• Phase I/II Open-label, Non-Randomized, Multi-Center Study	• 0 patients	• SVF harvested from Autologous Adipose Tissue will be deliver after processing via IV and Inhalation	None	• Primary outcomes: Functional Capacity improved compared to baseline [Time Frame: 3 months, 6 months], Number of adverse events [Time Frame: 3 months, 6 months]	Withdrawn (company dissolved)
					• Secondary outcomes: Quality of Life improved compared to baseline [Time Frame: 3 months, 6 months].	
Cell Therapy in Advanced Chronic Obstructive Pulmonary Disease Patients (2015)	• Phase I/II randomized, open- label, placebo-control study	• 20 patients (COPD patients with persistent dyspnea in stage 2 or 3 of the dyspnea scale score; Eligibility for pulmonary rehabilitation program; No smoking or smoking cessation for at least 6 months, abscense of emphysema)	• BMMC: 1 × 10^8 B.M. in 30 mL saline IV.	No interventions will be performed other than conventional (in-course) treatment.	• Primary outcomes: Pulmonary morphology [Time Frame: 9 months after procedure]	Unknown
			•ASC: 1 × 10^8 ASC in 30 mL saline IV.			
			•BMMC + ASC: 5 × 10^7 ASC + 5 × 10^7 B.M. in 30 mL saline IV.		• Secondary outcomes: Pulmonary morphology [Time Frame: 9 months after procedure]; Pulmonary function [Time Frame: 12 months after procedure]	
Use of Autologous, Adult Adipose-Derived Stem/Stromal Cells In Chronic Lung Disorders (ADcSVF-COPD) (2016)	• Phase I/II non-randomized, single-blind, study	• 100 patients (18‒80 years, prior diagnosis of moderate to severe COPD; GOLD IIa, III, IV);no positve hepatites)	• Experimental: Isolation and IV administration of cellular stem/stromal cells from subdermal adipose-derived cellular stromal vascular fraction. Intervention: Procedure: SVF	None	• Primary outcomes: Safety ‒ Pulmonary Function [Time Frame: 12 months Evaluate Function and Adverse Events], Change from Baseline Respiratory Rate [Time Frame: 1 month, 6 month, 1 year].	Enrolling by invitation
			• Experimental: Normal Saline IV Arm 3 with SVF cells		• Secondary outcomes: GOLD Classification [Time Frame: 1 year]; Change from baseline 6 Min Walk Test [Time Frame: 12 Months]; Exercise capacity measured by distance a patient can walk in 6 min timeframe; Change from Baseline Lung X-Ray [Time Frame: 6 months, 12 months]; Change from Baseline SGOT Blood Testing [Time Frame: 1 Month]; Change from Baseline SGPT Blood Testing [Time Frame: 1 Month]; Pulmonary Function Testing [Time Frame: Baseline, 6 Months].	
Autologous Adipose-derived Stem Cells (AdMSCs) for COVID-19 (2020)	• Phase II randomized, double-blind, placebo-control study conducted in multiple clinic facilities	• 200 participants (> 18 years; male or female; have banked AdMSCs in Celltex; written informed consent; highly susceptible to SARS-CoV-2 infections, no terminal stages; no previous COVID-19 history, SARS-CoV-2 RT-PCR or equivalent tests negative; SARS-CoV-2 IgM and IgG negative)	• Three doses of 200 million autologous adipose derived mesenchymal stem cells via intravenously infusion every three days	• Three doses of placebo via intravenously infusion every three days.	• Primary outcomes: Assessment of the total number of AEs/SAEs related and non-related with the medication [Time Frame: 6 months]; Proportion of AEs/SAEs related and non-related with the ASCs infusions as compared to the control group [Time Frame: 6 months]; COVID-19 incidence rates [Time Frame: 6 months]	Not yet recruiting
					• Secondary outcomes: Proportion of SARS-CoV-2 infected subjects testing [Time Frame: 6 months]; Proportion of mild, classic, severe and critically sever symptomatic SARS-CoV-2 infected subjects [Time Frame: 6 months]; Change of proportion of SARS-Cov-2 infected subjects IgM/IgG + against SARS-CoV-2. [Time Frame: 6 months]; Change of lymps count from the baseline [Time Frame: 6 months]; Change of PaO2 from the baseline [Time Frame: 6 months]; Compare the proportion of severe COVID-19 pneumonia cases development [Time Frame: 6 months]; COVID-19 mortality rates [Time Frame: 6 months]; Change of CRP (mg/L), d-dimer (mg/L, Procalcitonin (ug)/L, pro-BNP (pg/mL), BI (mg/dL), Cr (mg/dL), from the baseline [Time Frame: 6 months]; Change in cytokine panels (IL-1β, IL-6, IL-8, IL-10, TNFα) from the baseline [Time Frame: 6 months]; Proportion of SARS-CoV-2 RT-PCR positive to negativity [Time Frame: 6 months]; Quantifying viral RNA in stool for baseline and final follow-up. [Time Frame: 6 months].	
Clinical Study for Subjects With COVID-19 Using Allogeneic Adipose Tissue-Derived Mesenchymal Stem Cells (AdMSCs) (2021)	• Phase II randomized, double blind and placebo controled study conducted initially in a single clinic facility.	• 30 participants (> 18 years; male or female; Diagnosed as COVID-19 based upon SARS-CoV-2 RT-PCR + test; Clinical diagnosis meets severe and/or critical parameters; Male participants must be willing to ensure their partners do not become pregnant either by practicing abstinence or the use of condoms during sexual activity)	• Three separate doses of 200 million allogeneic adipose-derived mesenchymal stem cells via intravenously infusion on days 0, 3, and 6 with a total of 600 million AdMSCs during 7 days in addition to their standard of care.	• The control group will receive placebo infusion on day 0, 3 and 6 along with standard of care.	• Primary outcomes: Frequency and nature of adverse events occurring during the study based on the rate of all ASC associated AEs in all subjects. [Time Frame: 6 months]; Safety for ASCs based upon incidence of all AEs [Time Frame: 6 months]; Comparison the mortality rate between treating group vs. control group [Time Frame: 6 months]	Not yet recruiting
					• Secondary outcomes: Change of SOFA score as compare to the baseline Time Frame: 6 months]; Organ functional tests including blood specific enzymes and proteins [Time Frame: 6 months]; Days of weaning from mechanical ventilation [Time Frame: 6 months]; Duration (days) of ICU monitoring [Time Frame: 6 months]; Duration (days) of vasoactive agent's usage [Time Frame: 6 months]; Days of hospitalization [Time Frame: 6 months]; Proportions of SARS-CoV-2 RT-PCR change to negative from respiratory tract specimens using CDC standard method [Time Frame: 6 months]; Proportions of quantifying viral RNA in stool change to negative in final follow-up using CDC standard method [Time Frame: 6 months]; Proportions of blood SARS-CoV-2 antibodies IgM/IgG show positive [Time Frame: 6 months].	
Clinical Study of Adipose Derived Mesenchymal Stem Cells for Treatment of Pulmonary Arterial Hypertension (2019)	• Phase I/II, randomized, double masked, parallelly assignmented study	• 60 participants (40‒75years; male or female; COPD with moderate to severe pulmonary hypertension; lifetime > 6 months; signed the informed consent in person)	• The MSCs of 1 × 10×6/kg will be given in Central venous catheterization for injection at a total 100 mL. The injection cycle was once every week of two times.	• Conventional drug therapy (expectorant, bronchodilator)	• Primary outcomes: Change in Pulmonary Vascular Resistance from Baseline [Time Frame: Baseline, 4, 12 and 24 weeks]	Unknown
					• Secondary outcomes: Change from Baseline in Participant Quality of Life Using the ASCs [Time Frame: Baseline, 4, 12 and 24 weeks]; Change in Plasma NT-pro-BNP levels [Time Frame: Baseline, 4, 12 and 24 weeks]; Change in the IL-1β, IL-6, PGE-2, TGF-β, TNF-α and IGF-1 (ng/ul) [Time Frame: Baseline, 4, 12 and 24 weeks]; Incidence of Treatment Adverse [Time Frame: Baseline, 4, 12 and 24 weeks]; Change in Six Minute Walk distance [Time Frame: Baseline, 4, 12 and 24 weeks]	
Evaluate Safety and Efficacy of Intravenous Autologous ADMSc for Treatment of Idiopathic Pulmonary Fibrosis (2014)	• Phase I/II, Prospective, Multicentric, Open Label, Randomized, Interventional Study	• 60 participants (40‒75years; male or female; COPD with moderate to severe pulmonary hypertension; lifetime > 6 months; signed the informed consent in person)	• Single dose of SVF IV;	• CCO ≤10 mg/day or ≤20 mg alternating days + Immunosuppressants 2 mg/kg/day, not exceeding 150 mg/day + Antioxidants up to 1800 mg/day + Pirfenidone up to 1200 to 1800 mg/day.	• Primary outcomes: Incidence of treatment emergent AEs in the study [Time Frame: 9 Month].	Unknown
			• 3 IV doses of 2 million/kg ASCs each, given at weekly intervals.			
					• Secondary outcomes: Change in predicted FVC% at EOS [Time Frame: 9 Month]; Change in predicted DLCO% at EOS [Time Frame: 9 Month]; Change in the 6MWT at EOS [Time Frame: 9 Month]; Changes in the disease extent and severity as reflected by HRCT (64 SLICE) at EOS from randomization [Time Frame: 9 Month].	
Study of Intravenous Administration of Allogeneic Adipose Stem Cells for COVID-19 (CoronaStem1) (2020)	• Phase I, open label, single group comparison with cohort of contemporaneous non-treated patients.	• 10 participants (Admitted to hospital as inpatient; respiratory distress; bilateral lung infiltrates; supplemental oxygen started but NOT intubated or ventilated; COVID-19 positive antigen test; time from enrollment to treatment < 24 h; age: 18‒80 years; gender: any; suitability for cellular therapy; preserved cognitive function)	• Adipose stem cells derived from screened donor lipoaspirate and culture expanded. The dosage was not described.	None	• Primary outcomes: Frequency of all AEs [Time Frame: Through study completion, an average of three months]; Frequency of infusion related SAEs [Time Frame: 6 h post infusion]; Frequency of SAEs [Time Frame: Through study completion, an average of three months];	Completed
					• Secondary outcomes: Mortality [Time Frame: Study days 0‒28]; Ventilator Free Days [Time Frame: Study days 0‒28]; ICU Free Days [Time Frame: Days 0 through 28]; Total Hospital Days [Time Frame: Days 0 through discharge, an average of 28 days]; Total ICU Days [Time Frame: Days 0 through discharge, an average of 28 days]; Improvement in Oxygenation [Time Frame: Study days 0, 2, 4, 6]	
Adipose-derived Mesenchymal Stem Cells in Acute Respiratory Distress Syndrome (2013)	• Phase I, randomized, triple blinded, parallelly assignmented study.	• 20 participants (ARDS diagnosed using Berlin definition; at least 18 years of age; acute onset of ARDS; Bilateral opacities in chest radiography; No cardiac failure; PaO2/FiO2 ratio < 200)	• One dose of 1 × 10^6 allogeneic adipose-derived mesenchymal stem cells/kg body weight intravenously within 48 h of enrollment.	• One dose of Intravenous saline infusion	• Primary outcomes: Compare the adverse events between mesenchymal stem cell treatment and placebo groups [Time Frame: From day 0 at the start of treatment to day 28].	Unknown
					• Secondary outcomes: Hospital indices by treatment group [Time Frame: From admission to discharge]	
Study of Allogeneic Adipose-Derived Mesenchymal Stem Cells to Treat Post COVID-19 "Long Haul" Pulmonary Compromise (2021)	• Phase IIa, randomized, open-label, parallelly assignmented study.	• 0 participants	• IV iASCs (∼18.5 million cells) on Day 0, Day 2, and Day 4.	None	• Primary outcomes: Change in 6MWD at Day 60 [Time Frame: Baseline to Day 60];	Withdrawn (Replaced by a different protocol.)
			• IV ASCs (∼37 million cells) on Day 0, Day 2, and Day 4.		• Secondary outcomes: Change in 6MWD at Day 30 [Time Frame: Baseline to Day 30]; Change in Pulmonary Function Tests (PFTs) [Time Frame: Baseline to Day 30 and Day 60]; Change in oxygenation [Time Frame: Baseline to Day 30 and Day 60]; Change in biomarker levels [Time Frame: Baseline through Day 30]	
A Clinical Trial to Determine the Safety and Efficacy of Hope Biosciences Autologous Mesenchymal Stem Cell Therapy (HB-adMSCs) to Provide Protection Against COVID-19 (2020)	• Phase II, Open Label, Single-Center, Clinical Trial	• 56 participants (Men, and women > 65 years OR works in high-risk environment OR has underlying conditions; have previously banked their cells at Hope Biosciences; no signs or symptoms of infection, subject provides written informed consent; agrees to the collection of venous blood per protocol.)	• Five IV infusions of autologous, adipose-derived mesenchymal stem cells.	None	• Primary outcomes: Incidence of hospitalization for COVID-19 [Time Frame: Week 0 through week 26]; Incidence of symptoms for COVID-19 [Time Frame: week 0 through week 26]	Completed
					• Secondary outcomes: Absence of upper/lower respiratory infection [Time Frame: Weeks 0 through 26]; Glucose, Ca, Albumin, Total proteína, Na, total carbono dioxide, Cl, AP, total BI, BUN, ALT,AST, Cr,Ht, MCHb, CHb, MCV, Platelets, CRP [Time Frame: Weeks 0, 6, 14, 26]; White blood cells [Time Frame: Weeks 0, 6, 14, 26]; Red blood cells [Time Frame: Weeks 0, 6, 14, 26]; Hb [Time Frame: Weeks 0, 6, 14, 26]; Ht [Time Frame: Weeks 0, 6, 14, 26];; Red cell distribution width [Time Frame: Weeks 0, 6, 14, 26]; Neutro [Time Frame: Weeks 0, 6, 14, 26]; Lymphs, Eos, Mono, Baso, [Time Frame: Weeks 0, 6, 14, 26]; Absolute neutro, Absolute lymphs, Absolute mono, Absolute eos, Absolute baso [Time Frame: Weeks 0, 6, 14, 26]; Immature granulocytes [Time Frame: Weeks 0, 6, 14, 26]; Absolute Immature granulocytes [Time Frame: Weeks 0, 6, 14, 26]; Prothrombin time [Time Frame: Weeks 0, 6, 14, 26]; INR [Time Frame: Weeks 0, 6, 14, 26]; TNFalpha [Time Frame: Weeks 0, 6, 14, 26]; IL-6 and IL-10 [Time Frame: Weeks 0, 6, 14, 26]; SF-36 [Time Frame: Weeks 0, 6, 14, 26]; PHQ-9 [Time Frame: Weeks 0, 6, 14, 26]	
Study of Allogeneic Adipose-Derived Mesenchymal Stem Cells for Non-COVID-19 Acute Respiratory Distress Syndrome (2021)	• Phase IIa Randomized, Placebo-Controlled Study	• 0 participants	• ASCs IV (two vials or a total of ≈30 million cells) on Day 0, Day 2, and Day 4	• Placebo IV (two vials) on Day 0, Day 2, and Day 4	• Primary outcomes: All-cause mortality rate at Day 28 [Time Frame: Baseline to Day 28];	Withdrawn (Replaced by a different protocol.)
					• Secondary outcomes: All-cause mortality rate at Days 60 and 90; Number of ventilator-free days through Day 28; Number of ICU days through Day 28; Clinical status at Day 28; Change in oxygenation [Time Frame: Baseline to Day 2, Day 4, Day 6, Day 14, Day 28].	
Study of Allogeneic Adipose-Derived Mesenchymal Stem Cells to Treat Post COVID-19 "Long Haul" Pulmonary Compromise (BR) (2021)	• Phase IIa Randomized, Placebo-Controlled study	• 60 participants (prior laboratory-confirmed SARS-CoV-2 infection; < 1 week negative SARS-CoV-2 test; at least moderate or severe post-COVID-19 pulmonary symptoms for at least 3 months which have resulted in reduced physical functioning compared to pre-COVID-19 status; willing to follow contraception guidelines).	• 2, 4 or 6 MSC vials IV (approximately 15million cells/vial) on Day 0, Day 2, or Day 4 depending on assignment to treatment group: Group A: 2 MSC vials infused on D0 and 2 vials of placebo on D2 and D4; Group B: 2 MSC vials infused on D0 and D2 and 2 vials of placebo on D4; Group C: 2 MSC vials infused on D0 and D4 and 2 vials of placebo on D2; Group D: 2 MSC vials infused on D0, D2 and D4	• 6 vials of placebo will be intravenously infused on Day 0, Day 2, or Day 4.	• Primary outcomes: Change in 6MWD at Day 60 [Time Frame: Baseline to Day 60]	Not yet recruiting
					• Secondary outcomes: Change in 6MWD at Day 30 [Time Frame: Baseline to Day 30]; Relief of symptoms on Day 30 and Day 60 [Time Frame: Baseline to Day 30 and Day 60]; Change in Pulmonary Function [Time Frame: Baseline to Day 30 and Day 60]; Change in oxygenation [Time Frame: Baseline to Day 30 and Day 60]; Change in biomarker levels [Time Frame: Baseline to Day 60]	
Study of Allogeneic Adipose-Derived Mesenchymal Stem Cells for Treatment of COVID-19 Acute Respiratory Distress (2021)	• Phase II, Randomized, parallelly assignmented, quadruple blinded study	• 60 participants (prior laboratory-confirmed SARS-CoV-2 infection; < 1 week negative SARS-CoV-2 test; hospitalized with at least "severe" COVID-19-induced ARD or ARDS; requires oxygen supplementation at Screening; willing to follow contraception guidelines).	• ASCs IV (two vials or a total of ≈ 30 million cells) on Day 0, Day 2, and Day 4	• Placebo IV (two vials) on Day 0, Day 2, and Day 4	• Primary outcomes: All-cause mortality rate at Day 28; Incidence of all adverse events (AEs) [Time Frame: Baseline through study completion at Day 90]; Incidence of treatment-emergent adverse events [Time Frame: Baseline through study completion at Day 90]; Incidence of severe adverse events [Time Frame: Baseline through study completion at Day 90]; Incidence of infusion-related adverse events [Time Frame: Baseline to Hour 4]	Recruiting
					• Secondary outcomes: All-cause mortality rate at Day 60 and 90; Number of ventilator-free days through Day 28; Number of ICU days through Day 28; Change in clinical status [Time Frame: Baseline to Day 28]; Change in clinical status as assessed using the WHO Clinical Progression Scale (0‒10 scale, where lower score means a better outcome) at Day 28; Change in oxygenation [Time Frame: Baseline to Day 14 Day 28, and Day 60].	
Clinical Study to Assess the Safety and Preliminary Efficacy of HCR040 in Acute Respiratory Distress Syndrome (2020)	• Phase I (open label) study and Phase II, randomized, controlled, double-blinded study	• PI: 6 participants with moderate to severe ARDS will be included in 2 sequential cohorts.	• PI: Open label IV dose escalation, 3 patients in cohort 1 (1 million cells/kg) and 3 patients in cohort 2 (2 million cells/kg)	• PI: None	• Primary outcomes: Number of AEs [Time Frame: One year];	Recruiting
		• PII: 20 participants with moderate to severe ARDS will be randomly divided into two groups (control and treated).	• PII: Maximum tolerated dose IV (1 million cells/kg or 2 million cells/kg).	• PII: IV vehicle solution.	• Secondary outcomes: Average stay in the ICU 28 days after the administration of HCR040; SOFA index at 3, 7, 14, 21, and 28 days after the administration of HCR040; Mechanical ventilation-free days 28 days after the administration of HCR040; Percent mortality 28 days after the administration of HCR040; Daily pulmonary mechanics values (Ppl, DP, CRS) [Time Frame: One year]; Determination of lung damage using the Murray scale at day 3, 7, 14 and 28 after the administration of HCR040; Vasopressor-free days 28 days after the administration of HCR040; ICU-free days 28 days after the administration of HCR040	
Study of Intravenous Administration of Allogeneic Adipose-Derived Mesenchymal Stem Cells for COVID-19-Induced Acute Respiratory Distress (2021)	• Phase II, Randomized, parallelly assignmented, double blinded study	• 0 participants	• 1 × 10^6 MSCs/kg or 1.5 × 10^6 MSCs/kg, depending on CRP level	• Equivalent volume of placebo will be administered	• Primary outcomes: Mortality at Day 28;	Withdrawn (Replaced by a different protocol.)
					• Secondary outcomes: Mortality at Days 60 and 90; Number of ventilator-free days [Time Frame: Randomization through Day 28]; Improvement in oxygenation [Time Frame: Randomization to Day 2, Day 4, Day 6, Day 14, Day 28]; SOFA score at Day 28.	
A Randomized, Double-Blind, Placebo-Controlled Clinical Trial to Determine the Safety and Efficacy of Hope Biosciences Allogeneic Mesenchymal Stem Cell Therapy (HB-adMSCs) to Provide Protection Against COVID-19 (2020)	• Randomized, Double-Blind, Placebo-Controlled Single-Center Clinical Trial	• 55 participants (all gender, > 18 years, high-risk potential exposure to COVID-19 job,no signs or symptoms of infection, agrees to the collection of venous blood per protocol, agrees to conformational testing for SARS-CoV-2 before end of study.	• 5 intravenous infusions of ASCs at 200 million cells/dose each. Infusions will occur at weeks 0, 2, 6, 10, and 14.	• 5 intravenous infusions of placebo intervention (saline). Infusions will occur at weeks 0, 2, 6, 10, and 14.	• Primary outcomes: Incidence of hospitalization for COVID-19 [Time Frame: week 0 through week 26]; Incidence of symptoms associated with COVID-19 [Time Frame: week 0 through week 26]	Completed
			• 5 intravenous infusions of100 million cells/dose each. Infusions will occur at weeks 0, 2, 6, 10, and 14.			
			• 5 intravenous infusions of 50 million cells/dose each. Infusions will occur at weeks 0, 2, 6, 10, and 14.,		• Secondary outcomes: Absence of upper/lower respiratory infection [Time Frame: week 0 through week 26]; Leukocyte differential [Time Frame: weeks 0, 6, 14, 26]; CRP [Time Frame: weeks 0, 6, 14, 26]; TNF alpha [Time Frame: weeks 0, 6, 14, 26]; IL-6 [Time Frame: weeks 0, 6, 14, 26]; IL-10 [Time Frame: weeks 0, 6, 14, 26]; Glucose [Time Frame: weeks 0, 6, 14, 26]; Ca [Time Frame: weeks 0, 6, 14, 26]; Albumin [Time Frame: weeks 0, 6, 14, 26]; Total protein [Time Frame: weeks 0, 6, 14, 26]; Na: [Time Frame: weeks 0, 6, 14, 26]; Total carbon dioxide [Time Frame: weeks 0, 6, 14, 26]; K [Time Frame: weeks 0, 6, 14, 26]; Cr [Time Frame: weeks 0, 6, 14, 26]; BUN [Time Frame: weeks 0, 6, 14, 26]; Cr[T*ime* F*rame: weeks* 0, 6, 14, 26]; AP [Time Frame: weeks 0, 6, 14, 26]; ALT [Time Frame: weeks 0, 6, 14, 26]; Total BI [Time Frame: weeks 0, 6, 14, 2]; white blood cells [Time Frame: weeks 0, 6, 14, 26]; red blood cells [Time Frame: weeks 0, 6, 14, 26]; Hb [Time Frame: weeks 0, 6, 14, 26]; Ht [Time Frame: weeks 0, 6, 14, 26]; MCV [Time Frame: weeks 0, 6, 14, 26]; MCHb [Time Frame: weeks 0, 6, 14, 26]; MCHb concentration [Time Frame: weeks 0, 6, 14, 26]; red cell distribution width [Time Frame: weeks 0, 6, 14, 26]; neutro [Time Frame: weeks 0, 6, 14, 26]; Lymphs [Time Frame: weeks 0, 6, 14, 26]; Mono [Time Frame: weeks 0, 6, 14, 26]; Eos [Time Frame: weeks 0, 6, 14, 26]; Baso [Time Frame: weeks 0, 6, 14, 26]; Absolute neutro [Time Frame: weeks 0, 6, 14, 26]; Absolute lymphs [Time Frame: weeks 0, 6, 14, 26]; Absolute mono [Time Frame: weeks 0, 6, 14, 26]; Absolute eos [Time Frame: weeks 0, 6, 14, 26]; Absolute baso [Time Frame: weeks 0, 6, 14, 26]; Immature granulocytes [Time Frame: weeks 0, 6, 14, 26]; Platelets [Time Frame: weeks 0, 6, 14, 26]; PTT [Time Frame: weeks 0, 6, 14, 26]; INR [Time Frame: weeks 0, 6, 14, 26]; SF-36 [Time Frame: weeks 0, 6, 14, 26]; PHQ-9 [Time Frame: weeks 0, 6, 14, 26]	
Clinical Trial to Assess the Safety and Efficacy of Intravenous Administration of Allogeneic Adult Mesenchymal Stem Cells of Expanded Adipose Tissue in Patients With Severe Pneumonia Due to COVID-19 (2020)	• Phase I / II Clinical Trial, Multicenter, Randomized and Controlled, Safety and Efficacy study	• 26 participants (Age ≥18, Clinical diagnosis of Pneumonia, severe or critical, caused by COVID-19 infection. Life expectancy > 48 h, Commitment to use a contraceptive method of proven efficacy in both men and women during the duration of the clinical trial.).	•Two doses of 80 million adipose-tissue derived mesenchymal stem cells	No intervention	• Primary outcomes: Safety of the administration of allogeneic mesenchymal stem cells derived from adipose tissue assessed by Adverse Event Rate [Time Frame: 12 months]; Efficacy of the administration of allogeneic mesenchymal stem cells derived from adipose tissue assessed by Survival Rate [Time Frame: 28 days]	Completed
Efficacy and Safety Study of Allogeneic HB-adMSCs for the Treatment of COVID-19 (2020)	• Phase II Randomized, Placebo-Controlled, Double-Blind, Efficacy and Safety Study	• 53 participants (Men, and women, > 18 years of age inclusively, Patient is hospitalized due to suspected COVID-19 infection, Agrees to the collection of venous blood per protocol).	• 4 IV infusions of HB-adMSCs at 100 million cells/dose. HB-adMSC infusions will occur at day 0, 3, 7, and 10.	• 4 IV infusions of placebo (saline solution). Infusions will occur at day 0, 3, 7, and 10.	• Primary outcomes: IL-6, CRP, Oxygenation, TNF alpha, IL-10 [Time Frame: screening, day 0, 7. 10]; Return to room air (RTRA) [Time Frame: Day 0, 3, 7, 10, 28].	Terminated (No need to continue with vaccine available)
					• Secundary outcomes: EKG qt interval, Leukocyte differential, Glucose, Ca, Albumin, Total protein, Na, Total carbon dioxide, K, Cl, BUN, Cr, AP, ALT, Total BI, White blood cells, Red blood cells, Hb, Ht, MCV, MCHb,MCHbC, Red celldistribution width, Neutro, Lymphs, Mono, Eos, Baso, Absolute neutro, Absolute lymphs, Absolute mono, Absolute eos, Absolute baso, Immature granulocytes, PTT, INR, NK cell surface antigen (CD3-CD54+), clinical lab evaluation of percentage of cells CD3- and CD54+ (%), CD4+/CD8+ ratio Myoglobin, Troponin, Creatinine kinase MB, Serum ferritin [Time Frame: screening, day 0, 7, 10]; Adverse events [Time Frame: screening through day 28]; 7-point ordinal scale [Time Frame: screening, day 0, 3, 7, 10, 28]; d-dimer [Time Frame: screening, day 0, 7, 10]; Chest X-Ray [Time Frame: Day 0, Day 28]; CT scan [Time Frame: Day 0, Day 28]; PCR test for SARS-CoV-2 [Time Frame: day 0, 3, 7, 10]	
Study of Intravenous COVI-MSC for Treatment of COVID-19 ‒ Induced Acute Respiratory Distress (2021)	• Phase II, Randomized, parallelly assignmented, quadruple blinded study	• 100 participants (Men, and women, > 18 years Laboratory-confirmed SARS-CoV-2 infection, Hospitalized with COVID-19-induced ARD or ARDS with a PaO2/FiO2 ≤300; Requires oxygen supplementation at Screening; Willing to follow contraception guidelines	• IV infusions of COVI-MSC (two vials or a total of ≈30 million cells) on Day 0, Day 2, and Day 4	• IV infusions of placebo (two vials) on Day 0, Day 2, and Day 4	• Primary outcomes: All-cause mortality rate at Day 28 [Time Frame: Baseline through Day 28];	Recruiting
					• Secundary outcomes: All-cause mortality rate at Day 60 and Day 90 [Time Frame: Baseline through Day 60 and Day 90]; Number of ventilator-free days through Day 28 [Time Frame: Baseline through Day 28]; Number of ICU days through Day 28 [Time Frame: Baseline through Day 28]; Change in clinical status [Time Frame: Baseline to Day 28]; Change in oxygenation [Time Frame: Baseline to Day 2, Day 4, Day 6, Day 14 and Day 28]	
Randomized Double-Blind Phase 2 Study of Allogeneic HB-adMSCs for the Treatment of Chronic Post-COVID-19 Syndrome (HBPCOVID02) (2021)	• Phase II, Randomized, Double-blinded, Single-center, Efficacy, and Safety Study	• 80 participants (Men, and women, 18‒70 years, proof of Post COVID-19 Syndrome in their medical records, diagnosed with Chronic post-COVID-19 syndrome for at least twelve weeks before, one or more neurological symptoms, participants should not be pregnant or plan to become pregnant during study participation and six months after the last investigational product administration, If their sexual partners can become pregnant, male participants should use a method of contraception during study participation and for six months after the last administration of the experimental drug, The study participant is able and willing to comply with the requirements of this clinical trial.	• ASCs (Does not describe the dosage)	• Sterile Normal Saline	• Primary outcomes: Changes in Visual Analog Scale of Neurological Symptoms. - Extreme fatigue, Changes in Visual Analog Scale of Neurological Symptoms. ‒ Brain fog, Changes in Visual Analog Scale of Neurological Symptoms. ‒ Headache, Changes in Visual Analog Scale of Neurological Symptoms. ‒ Sleep disturbances, Changes in Visual Analog Scale of Neurological Symptoms. ‒ Loss of taste, Changes in Visual Analog Scale of Neurological Symptoms. ‒ Loss of smell, Incidence of treatment-emergent Adverse Event (TEAEs), Incidence of treatment-emergent Serious Adverse Events (SAEs), AEs of special interest (serious or non-serious) ‒ thromboembolic events, AEs of special interest (serious or non-serious) ‒ thromboembolism of the extremities. [Time Frame: Baseline to Weeks 26]. Incidence and risk of AEs of special interest (serious or non-serious), including peripheral events defined as, thromboembolism of the extremities, AEs of special interest (serious or non-serious) – infections, Incidence and risk of AEs of special interest (serious or non-serious), including infections, AEs of special interest (serious or non-serious) – hypersensitivities, Changes in Laboratory values. – CBC, Changes in Laboratory values. – CMP, Changes in Laboratory values. ‒ Coagulation Panel, Changes in Vital Signs. ‒ Respiratory Rate (breaths per minute), Changes in Vital Signs. ‒ Heart Rate (beats per minute), Changes in Vital Signs. ‒ Body Temperature (Fahrenheit), Changes in Vital Signs. ‒ Blood Pressure (mmHg), Changes in Weight in lb., Changes in Physical examination results. ‒ General [Time Frame: Baseline to Weeks 26], Clinically significant changes in general physical examination results. Changes in Physical examination results. ‒ Body Systems [Time Frame: Baseline to Weeks 26]	Recruiting
					• Secundary outcomes: Changes in Subject's energy ‒ Fatigue Assessment form, Changes in Visual Analog Scale of non-Neurological Symptoms. ‒ Dyspnea a rest, Changes in Visual Analog Scale of non ‒ Neurological Symptoms. ‒ Dyspnea with activity, Changes in Visual Analog Scale of non ‒Neurological Symptoms. – Cough, Changes in Visual Analog Scale of non ‒ Neurological Symptoms. ‒ Body aches, Changes in Visual Analog Scale of non-Neurological Symptoms. ‒ Joint pain, Changes in Subject's quality of life ‒ Short Form 36 Health Survey Questionnaire, Changes in Subject's level of depression ‒ PHQ 9 scale. [Time Frame: Baseline to Weeks 26]	
BAttLe Against COVID-19 Using Mesenchymal Stromal Cells (2020)	• Phase II Two-treatment,Randomized, Controlled, Multicenter Clinical Trial	• 80 participants (Men,women, 18‒70 years, proof of Post COVID-19 Syndrome, participants should not be pregnant or plan to become pregnant during study participation and six months after the last investigational product administration, If their sexual partners can become pregnant, male participants should use a method of contraception during study participation and for six months after.	• Two serial doses of 1.5 million adipose-tissue derived mesenchymal stem cells per kg	• Regular respiratory distress treatment	• Primary Outcomes: Efficacy of the administration of allogeneic mesenchymal stem cells derived from adipose tissue assessed by Survival Rate) [Time Frame: 28 days]; Safety of the administration of allogeneic mesenchymal stem cells derived from adipose tissue assessed by Adverse Event Rate [Time Frame: 6 months]	Suspended (lack of financial support)
Intermediate Size Expanded Access Protocol for the Treatment of Post-COVID-19 Syndrome (2021)	• Does not describe study method or phase	• Does not describe number os participants	• Route: Intravenous	• Does not describe if there is a control group	• Does not describe the outcomes	No longer available
			• Dose: 200 million autologous adipose derived mesenchymal stem cells.			
Study to Evaluate the Efficacy and Safety of AstroStem-V in Treatment of COVID-19 Pneumonia (2020)	• Phase I/Ⅱa, open label, single group assignment, Trial to Explore the Safety and Efficacy study	• 10 participants (19‒80 years; diagnosed with pneumonia by radiologic examination, hospitalized for pneumonia caused by COVID-19 infection at screening, subject who has moderate COVID-19 disease, voluntarily participate in the clinical trial with written informed consent	• ASCs (Does not describe the dosage)	None	• Primary outcomes: Treatment related adverse events [Time Frame: From baseline to Week 12]; Number of subjects with treatment related abnormal variation of vital signs, physical examination and laboratory test values [Time Frame: From baseline to Week 12]	Not yet recruiting
					• Secondary outcomes: Oxygenation index (PaO2/FiO2 ratio) [Time Frame: From baseline to Week 12]; Mortality rate [Time Frame: Week 4, Week 8, and Week 12]; Ventilator treatment status [Time Frame: From Week 1 to Week 12]; Improvement of pneumonia [Time Frame: From baseline to Week 12]; SOFA [Time Frame: From baseline to Week 12]; 2019 nCOV nucleic acid test [Time Frame: From baseline to Week 12].	
Cx611–0204 SEPCELL Study (2020)	• Phase Ib/IIa, randomised, double-blind, multicentre trial.	• 84 patients with 18–80 years; body weight 50–100 kg; clinical diagnosis of sCABP (within ≤21 past days) + radiographic findings; ICU management, IMV or treatment with vasopressors for at least 2 h, negative pregnancy treatment.	• Two central line infusions of Cx611 administered within 3 days (on days 1 and 3) at a dose of 160 million cells each	• Will receive SoC therapy according to local guidelines plus two intravenous central line infusions of Ringer Lactate.	• Primary outcomes: safety profile and potential immunological host responses against the administered cells during the follow-up period.	Completed
			• Does not describe stem cell (CD) markers			
			•Follow up: up to day 730		• Secondary outcomes: explore the clinical efficacy of Cx611 in terms of a reduction of the duration of mechanical ventilation and/or the need for vasopressors and/or improved survival and/or clinical cure of the sCABP, as well as other efficacy-related endpoints.	

SVF, Stromal Vascular Fraction; PRP, Platelet Rich Plasma; BMMC, Bone Marrow Mononuclear Cells; ASCs, Adipose-derived Stem Cell; COPD, Chronic Obstructive Pulmonary Disease; CRP, C-Reactive Protein; Pro-BNP, Pro-type B Natriuretic peptide; BI, Bilirubin; Cr, Creatinine; AEs, Adverse Effects; SAEs, Severe Adverse Effects; SOFA, Sequential Organ Failure Assessment; IV, Intravenously; CCO, Corticosteroids; ARDS, Acute Espiratory Distress Syndrome; 6MWD, 6-Minute Walk Distance; AP, Alkaline Phosphatase; ALT,. Alanine Aminotransferase; AST, Aspartate Aminotransferase; K, Potassium; Hb, Hemoglobin; Ht, Hematocrit; MCV, Mean Corpuscular Volume; MCHb, Mean Corpuscular Hemoglobin; Eos, Eosinophils; Neutro, Neutrophils; Lymphs, Lymphocytes; Mono, Monocytes; Baso, Basophils; Ca, Calcium; Na, Sodium; Cl, Chloride; PTT, Prothrombin Time; SF-36, Short-Form 36 Health Survey.

Searching the gray literature did not present results contemplated by the subject of the study.

## Discussion

Although the mechanisms by which ASCs reduce lung inflammation and promote tissue repair are not fully elucidated [3], the use of mesenchymal stem cells in acute lung diseases had previously been reviewed by current literature showing promising results [13]. Since the initial analysis of the new disease caused by SARS-CoV-2 demonstrated main pathologic features similar to ALI/ARDS [14], the hypothesis of transposing these benefits in the context of a new pandemic without known therapeutic options were naturally investigated [1,3,14]. However, upon closer analysis, peculiarities were found in the pathophysiology of COVID-19 that benefited from autologous or allogeneic IV ASCs in a different way than those initially imagined [3].

In this context the present study proposed to analyze the benefits of cell therapy in COVID-19, exposing the possible common path among chronic and acute lung diseases that allow COVID-19 to manifest itself like chronic lung diseases [1,6], with fibrosis and pulmonary consolidation, but with an acute and fulminant evolution [6], owing to inflammatory exudation, pulmonary edema, and inflammatory cytokine storm.

Thus, the effectiveness evidenced by Liu et al. [3], Siu et al. [15]. and other studies is here revised as being due to immune dysregulation and fibrosis being common components of the pathophysiology of chronic and acute lung diseases, being closely related to their morbidity and mortality despite the different etiologies [7,13]. This convergence differs from a physiological immune response by inflammation resulting from both the activation of native pulmonary macrophages, molecular patterns associated with pathogens or associated damage, and the overproduction of alarmins that attract circulating immune cells to the lungs, initiating inflammation secondary to trauma and hypersensitivity [16,17].

Regarding clinical parameters, the present review is in line with similar studies by showing that IV administration of ASCs: has pulmonary homing, rescued the suppressive effects of cigarette smoke on bone marrow hematopoietic progenitor cell function [18], restored sustained weight loss [8,18,19], reduced PF score [8,19], increased survival in animal models improved the PF Ashcroft score [8,19], attenuated pulmonary edema [18,20], preserved pulmonary architecture [8,19,21,22,23], reduced allergic symptoms and mucus production [20,22], in addition to exerting protective effects on ALI secondary to pulmonary infection by *P. aeruginosa* [24,25,26].

In opposition to the study by Feizpour et al. [27], the histopathological endpoints showed that ASC IV, not only reduced inflammatory infiltration 28, 29, 30, 31, decreased lung cell death 19, 31, 32, 33, 34 and increased air space [35,36], but also attenuated the increase in inflammatory cells 28, 29, 30, 31 and presented tissue regenerative potential 31, 32, 33.

These findings are most likely due to the remodeling capacity of the microenvironment exhibited by ASCs IV 31, 37, 38 through antioxidant and anti-apoptotic properties by inhibiting IL-4, IL-5, and IL-13 from the Th2 pathway concomitant with the increase in Th1 cytokines 11, 12, 31, 37, 38. Furthermore, ASCS decreased levels of TGF-β, collagen I fibers, apoptotic cells, plasma fibrinogen, PDGF, Von Willebrand factor, NOS-2, FGF7, CC16, CK19, myeloperoxidase, MIP-2 and proteins totals in BALF 13, 18, 19, 20, 21, 22, 39 as well as inhibited: total immune cells, NET formation, fibroblast activation, collagen deposition, epithelial-mesenchymal transition, bacterial loads, iNOS, NFкB and Caspase-3 expression; in addition to significantly increasing the Bcl-2/Bax ratio [24, 25, 26, 27, 28,30,35,40, 41, 42].

Unlike similar studies that did not review the dosing regimen used, nor its effect on the studied endpoints, the present systematic review suggests that the fastest dose-dependent effect was exerted by cells cryopreserved at the primary site of infection [27] and the high dose showed not only a greater decrease in these parameters but also a low expression of αSMA and reversal of induced histopathological changes [26,43,44].

Therefore, and in accordance with other similar studies, this review suggests: the safety of IV ASCs 39, 43, 44, 45 31, 39, 43, 44, 45, based on the absence of serious adverse effects or toxicity to their administration, and the applicability of ASCs in ALIs of different pathophysiological mechanisms 5, 6, 14, 20, 23, 28, 29, 31, 37, 38, 39, including severe COVID-19 [1,6,26,40,43]. The physiological rationale reviewed suggests that therapy with ASCs can reduce lung damage in a patient with ARDS from SARS-CoV-2 infection, in addition to promoting leukocyte and lymphocyte recovery with its immunomodulatory and anti-apoptotic effects 12, 17, 26, 40, 43.

This study has among its limitations the selection bias, inherent to any non-systematic review; the limitation of most studies to interventions in the early inflammatory phase, offering better support for acute exacerbations to the detriment of its real applicability in the chronic fibrotic phase of the disease; the non-standardization of treatment time and dosage; as well as the lack of methodological rigor of some evidence included by not describing: their MSC surface markers, the parameters used in the analysis of the studies, nor the presence or absence of adverse effects.

Databases used in the present article are the main ones used in similar studies and allow contact with the vast amount of available literature on the subject. However, EMBASE database could not be included since CAPES periodicals does not provide its access through CAFe space. In addition, as it is a topic of recent emergence in the literature and, consequently, has an insufficient amount of clinical evidence for analysis, this study includes narrative reviews and preclinical studies to provide a summary of the currently available evidence on the topic, however, these study types have low-level certainty and high-level biases.

Finally, although the revised clinical data suggests optimism in the applicability of ASCs in other immunoinflammatory diseases 5, 6, 14, 15, 16, 17, 20, 21, 22, 23, 28, 29, 30, 31, 37, 38, 39, 40, 41, 42, 43 the little clinical evidence available about the effectiveness of this treatment lacks standardization, making it difficult to extrapolate its results. Therefore, further studies are needed to be focused on the elaboration of a consensus on the methods of collection of ASCs, the ideal dosage schedule, the most effective time and route of administration, as well as on the definition of indications for the administration of ASCs in cases of COVID-19 for conducting clinical trials soon.

## Conclusion

The revised clinical data suggests optimism in the applicability of ASCs in other immunoinflammatory diseases and in severe COVID-19 ARDS. However, further studies are needed to develop a consensus on the methods of collection of ASCs, the ideal dosage schedule, the most effective time and route of administration, as well as on the definition of indications for the administration of ASCs in cases of COVID-19 for conducting clinical trials in near future.

## Authors’ contributions

Bruna Benigna Sales Armstrong: Collected the data, performed the analysis and wrote the paper.

Juan Carlos Montano Pedroso: Supervised the project, revised it critically for important intellectual content and made a substantial contribution to the interpretation of data.

José da Conceição Carvalho Jr.: Supervised the project, revised it critically for important intellectual content and made a substantial contribution to the interpretation of data.

Lydia Masako Ferreira: Conceived and designed the review, supervised the project, revised it critically for important intellectual content, and gave the final approval of the version to be published. All authors reviewed the results and approved the final version of the manuscript

## Financial support and sponsorship

None.

## Declration of Competing Interest

The authors declare no conflicts of interest.
